# Theoretical study of the geometric and electronic characterization of carbendazim-based drug (Nocodazole)

**DOI:** 10.1016/j.heliyon.2020.e04055

**Published:** 2020-06-06

**Authors:** Muhammad Khattab

**Affiliations:** a Department of Chemistry of Natural and Microbial Products, Division of Pharmaceutical and Drug Industries, National Research Centre, Cairo, 12622, Egypt; b Department of Chemistry and Biotechnology, Faculty of Science, Engineering and Technology, Swinburne University of Technology, Melbourne, Victoria, 3122, Australia

**Keywords:** Physical chemistry, Theoretical chemistry, Pharmaceutical chemistry, Drug repurposing, DFT calculation, Benzimidazole carbamates

## Abstract

Scarcity in studies defining the precise three-dimensional structure of approved drugs has led to an abandoning of their use for other therapeutic indications. In this manuscript, we solely focus on studying computationally the anticancer drug “Nocodazole” as a model compound for anthelmintic drugs –due to structural similarity– proven to exert anticancer activity such as Mebendazole and Albendazole. Computations on Nocodazole structures deposited in the Protein Data Bank (PDB) revealed possible existence of at least 6 conformers of Nocodazole. By combining the reported experimental UV-Vis data with our calculations, two conformers were assigned as the predominant structures of Nocodazole. In addition, td-DFT calculations revealed that the conformational flexibility of Nocodazole results in significant changes in atomic and molecular charge densities. The results have ramifications in identification of possible conformers of carbendazim-based drugs for repurposing in oncology through giving deep insights in understanding the spatial and electronic changes upon drug binding to anticancer targets.

## 1 Introduction

Cancer is a group of diseases having high prevalence worldwide particularly in Australia. It accounts for more than a hundred of hardly curable illnesses [1]. Cancer is estimated responsible for 1-in-8 deaths globally. In Australia, cancer is also a leading cause of death across all age groups. It accounts for 34 per cent of the total fatal disease burden. In 2018, an estimated 138,321 Australians were diagnosed with cancer [2], excluding non-melanoma skin cancer. Based on recent national surveys of Australia, it is anticipated that 1-in-2 Australians will be diagnosed with cancer by the age of 85 [3]. The success of non-radiative cancer therapies is limited by the dynamic remodelling of cancerous cells which results in a poor efficacy of non-specific anticancer drugs. It also results in emergence of human cells resistant to cancer therapies [4]. Therefore, understanding the structure of a drug and when it binds to target receptor on the dynamic cancerous cells is crucial for developing new anticancer drug candidates and for understanding of cells resistance mechanism toward anticancer drugs as well.

Oncological drug development is a risky, exorbitant, and time-consuming process. The development time for anticancer drugs, from the time of the first filing to the granting of NDA/BLA approval, is estimated to range from 10 to 15 years [5]. The estimated success rate for new anticancer drugs from Phase I trial to FDA approval is around 6.7% over the period 2003 to 2011 [5]. However, patients threatened by cancer require a new and rapid development process of effective anticancer drugs. The emerging question then is, *can scientists accelerate oncological drug development? How?*

Drug repurposing in oncology, also called “Therapeutic Switching”, is the discovery and development of new anticancer drugs from drugs used for other non-anticancer indications. In contrary to the *de novo* drug discovery, repurposed anticancer candidates are spontaneously introduced to clinical trials speeding up their review by FDA, since its pharmacokinetics, pharmacodynamics and toxicity profiles in animals and humans have been previously studied and reported. Some benzimidazole-approved drugs -undergoing preclinical and clinical trials-for repurposing in chemotherapy are listed in Table 1. These drugs are placed in the WHO essential list of medicines indicating their safe use for the intended therapeutic indication [6]. Moreover, they are cost-effective drugs as the average cost of single dose is less than 0.1 USD. By studying the structure-function relationship of these drugs, cancer patients are anticipated to receive targeted, effective, safe, and cheap drugs for curing their ailments.

**Table 1:** Some benzimidazole-based drugs along with their trade names, classified according to their status by the World Health Organisation (WHO), biological target for the initial therapeutic indication, and cost per single dose.

	Mebendazole	Albendazole	Triclabendazole	Omeprazole	Bendamustine
Trade Name	Vermox®	Albenza®	Egaten®	Losec®	Treanda®
WHO Ess. Med.	Yes	Yes	Yes	Yes	Yes
Target	Parasitic worms	Parasitic worms	Parasitic worms	PPI	Leukemia, lymphoma, myeloma [7].
Anticancer Potential	CBCIs, kinase inh [8]. (colon, lung, adrenocortical, breast cancer) [9]	Modulation of oxidative stress, induction of DNA damage [10]. (colorectal, hepatoc. carcinoma)	Abcg2 inh [11].	FASN thioesterase activity inh. [12], colon cancer [13].	
Cost (USD)/Dose	0.04–0.004	0.06		0.01–0.06	0.06

To date, various benzimidazole-based drugs have been FDA-approved and are available as prescription drugs. Benzimidazole-based drugs are a proven class of molecules that exert broad therapeutic activities including anticancer (Nocodazole, Bendamustine), antiviral (Maribavir), anthelmintic (Mebendazole, Albendazole, Oxibendazole), antihistaminic (Astemizole, Mizolastine), antihypertensive (Candesartan, Telmisartan), fungicidal (Fuberidazole, Thiabendazole), and anticoagulant (Dabigatran) activities [14]. Therefore, benzimidazole ring system is an indispensable anchor for development of a broad range of pharmaceuticals [14].

Benzimidazol-2-carbamate, or carbendazim, is the scaffold of various anthelmintic drugs. This pharmacophore group was also found to exert anticancer activity such as in Nocodazole drug, introduced by Janssen Pharmaceutica. Despite of the therapeutic significance of carbendazim moiety, the tautomeric and conformational studies of carbendazim have not received much attention [15]. For instance, the mode of action of Nocodazole is believed to interfere with polymerization of microtubules, however the molecular mechanisms underlying its anticancer activities remain unclear.

**Figure 1:**
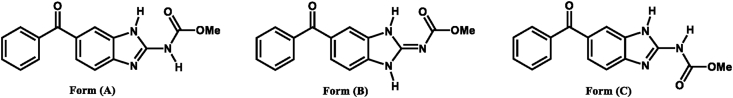
The 2D chemical structure of the three polymorphs of Mebendazole; reproduced from [19].

Nocodazole is a promising lead compound for the development of new anticancer drugs because of its sub-micromolar potency against both wild-type and mutant forms of Abl. Molecular dynamics studies of Nocodazole postulate a binding interaction between the aminobenzimidazole moiety of Nocodazole and the ATP binding sites of specific kinases [16]. Some of the anthelmintic drugs that share the carbendazim scaffold, such as Mebendazole and Albendazole, have been proven to exert antitumor activity as well. Mebendazole is an anthelmintic drug with over 40 years of safe use as an over the counter medication. It is listed as one of the World Health Organisation (WHO) essential medicines [6]. Mebendazole has been recently undergoing clinical trials in order to repurposing it for glioblastoma cancer therapy. It can also be used for treatment of other cancers, as well as a chemopreventive agent [17]. Discrepancies in its anthelmintic potency was reported due to the existence of different polymorphs [18]. Mebendazole can exist in three polymorphic forms - form A, B, and C (Figure 1) [19]. Form A was found to be therapeutically ineffective, while form C was reported as the most effective. Polymorph B is toxic due to its high aqueous solubility [20]. However, the relevance between different polymorphs and therapeutic anticancer activity is still not fully investigated. A recent study has revealed that polymorph C is a superior form as it targets brain tumors in effective concentrations [17].

Polymorphism is the ability of solid material to exist in more than one crystalline form with different arrangements or conformations of building block of the crystal lattice. Desmotropy (tautomeric polymorphism) is a rare phenomenon in which both tautomeric forms can been *isolated* in the solid state [21]. Polymorphic (tautomeric or conformational) forms of a drug not only differ in the physicochemical properties such as chemical and physical stability, dissolution, solubility, hygroscopicity, and flowability, but also differ in biological activity, drug efficacy, bioavailability, and toxicity. Therefore, structural elucidation studies and determination of relative polymorph content are important as a particular polymorph can account for a physicochemical property which might not be exhibited by another polymorph [22].

**Figure 2:**
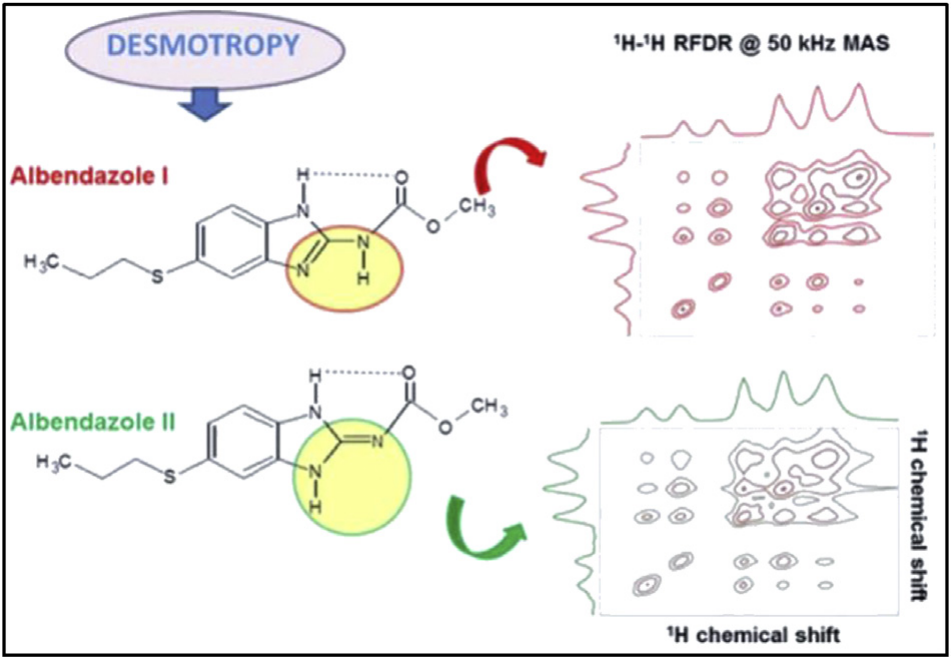
Structures of Albendazole desmotropes along with their corresponding ^1^H/^1^H fp-RFDR spectrum; reproduced from [23].

**Figure 3:**
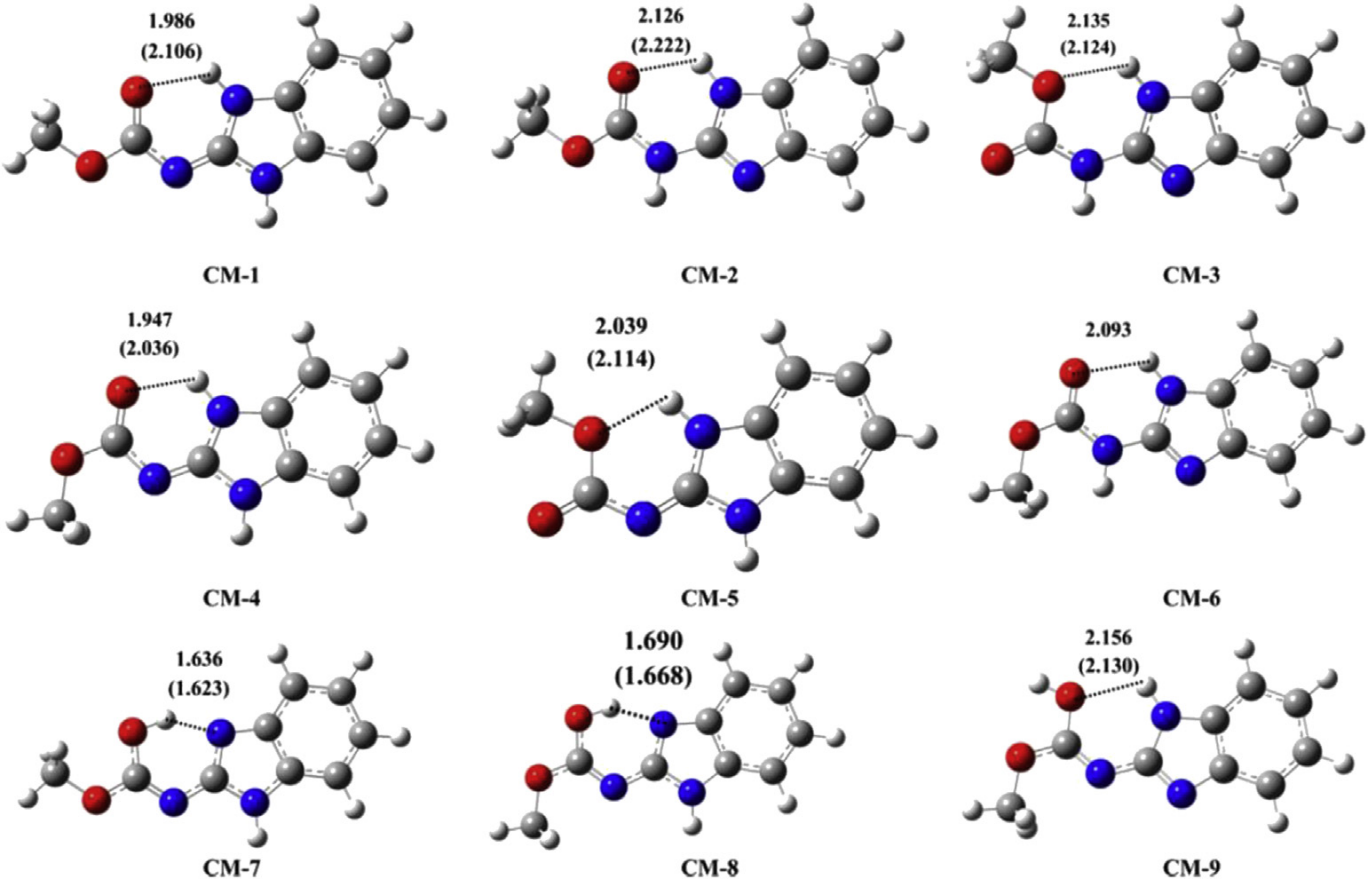
Optimized 3D geometries of carbendazim (CM-1 to CM-9) obtained using B3LYP/6-31+G(d) method. Dashed lines represent hydrogen bonds. Numbers and data in parentheses refer to H-bond length in gas and water medium respectively. Reproduced from [15].

For instance, Albendazole was found to exist in two distinct desmotropic structures, form I and II. The structural characterization of each desmotrope was obtained from the corresponding 2D-NMR and ^1^H/^13^C HETCOR spectrum which gave detailed information on the dimeric arrangements and intramolecular/intermolecular hydrogen bonding in two desmotropes of Albendazole [23]. A significant difference between the two forms was detected upon the assignment of the NH protons. In one form, the carbamate moiety is linked to the benzimidazole ring through amidic bond, while an imidic bond connects the two moieties in the other form (Figure 2) [23]. Referring to Kasetti and co-workers study, nine tautomeric structures of benzimidazol-2-carbamate have been proposed and studied theoretically (Figure 3) [15]. It was found that Albendazole form I corresponds to CM-2 structure, while the form II corresponds to CM-1 structure [15].

On the other hand, deoxyribose nucleic acid (DNA) bases contain benzimidazole nucleus where the tautomerism within DNA bases has been correlated with molecular-based diseases such as cancer [24]. The inference to be explored herein is that the tautomerism within benzimidazole-based drugs is expected to induce changes in their biological activity [25]. However, studies reporting the tautomeric populations of benzimidazoles have been overlooked, apparently due to the fast protropic exchange rate on benzimidazole nitrogens at room temperature [26]. Literature studies on benzimidazoles, almost exclusively, focused on synthetic methods and characterisation of benzimidazolyl derivatives [27, 28, 29]. Only a few researchers have discussed the tautomerism of benzimidazole compounds [24, 30], and most studies were aimed at obtaining well-resolved NMR spectra instead of studying the structural tautomerism itself. However, combining NMR spectroscopic measurements (as an experimental tool) with theoretical calculations has not received much attention in studying the structure of benzimidazole-based drugs in either the free or bound forms with target proteins, where different thermodynamic minima structures of the drug can be accessible [24, 30].

In this manuscript, we aim to theoretically study the structural and electronic configuration of the Nocodazole drug. To this end, we performed energy minimization calculations and time-dependent density functional calculations. In addition, a comparative theoretical study was performed between different structures of Nocodazole deposited in the Protein Data Bank (PDB) and our proposed structures. We also conducted analyses of some X-ray data deposited in PDB. Our study provides deep insights on the geometric and electronic changes in Nocodazole molecule paving the way for identification of different conformers of Nocodazole which can be used in drug repurposing models.

## 2 Methods

### 2.1 Computational details

Structure coordinates were downloaded from Protein Databank www.rcsb.org. The missing hydrogen atoms were added using Gaussview software. Three entries of Nocodazole along with the ideal structure were used with no further geometry optimization for performing computations. Time dependant density functional theory (TD-DFT) [31] was used for excitation energy calculation using the Becke three-parameters Lee-Yang-Parr hybrid functional (B3LYP) [32, 33] in combination with 6-311+G∗ basis set and conductor-like polarizable continuum model (CPCM) [34]. Dielectric constant of ε = 78.35 and 24.85 was used to approximately describe the polarity of bulk environment of water and ethanol respectively. The excitation (absorption) energies of Nocodazole structures were calculated for the singlet–singlet transitions of the lowest 45 excited states. All simulations were performed using GAUSSIAN 09 Revision C.01 [35] on Swinburne supercomputing facilities.

## 3 Results

### 3.1 Computational identification of Nocodazole conformers

**Figure 4:**
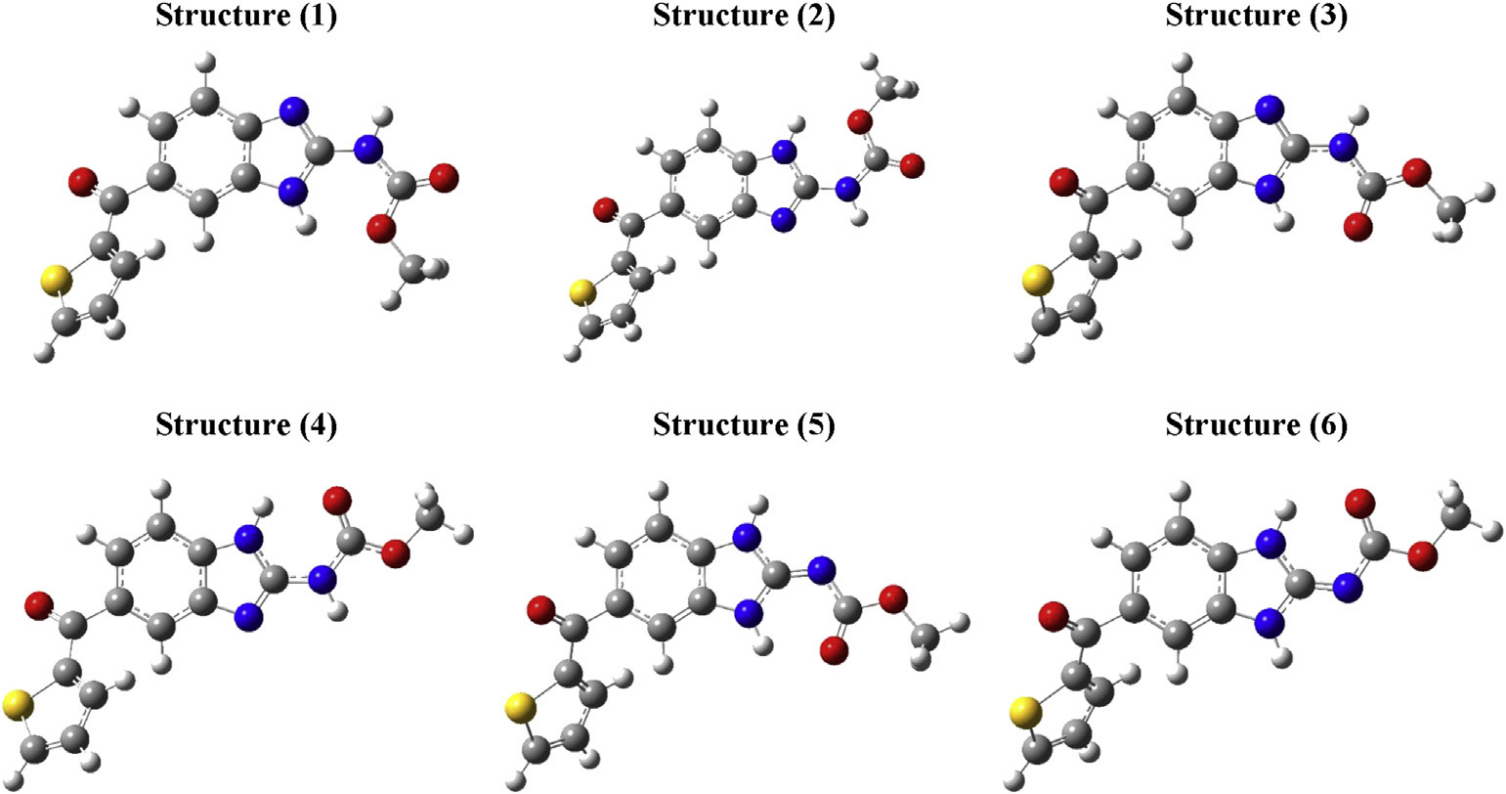
The 3D dimensional structures of the studied Nocodazole molecule calculated through energy minimization of different structures at B3LYP/6-311+G∗ level of theory in gas phase.

**Figure 5:**
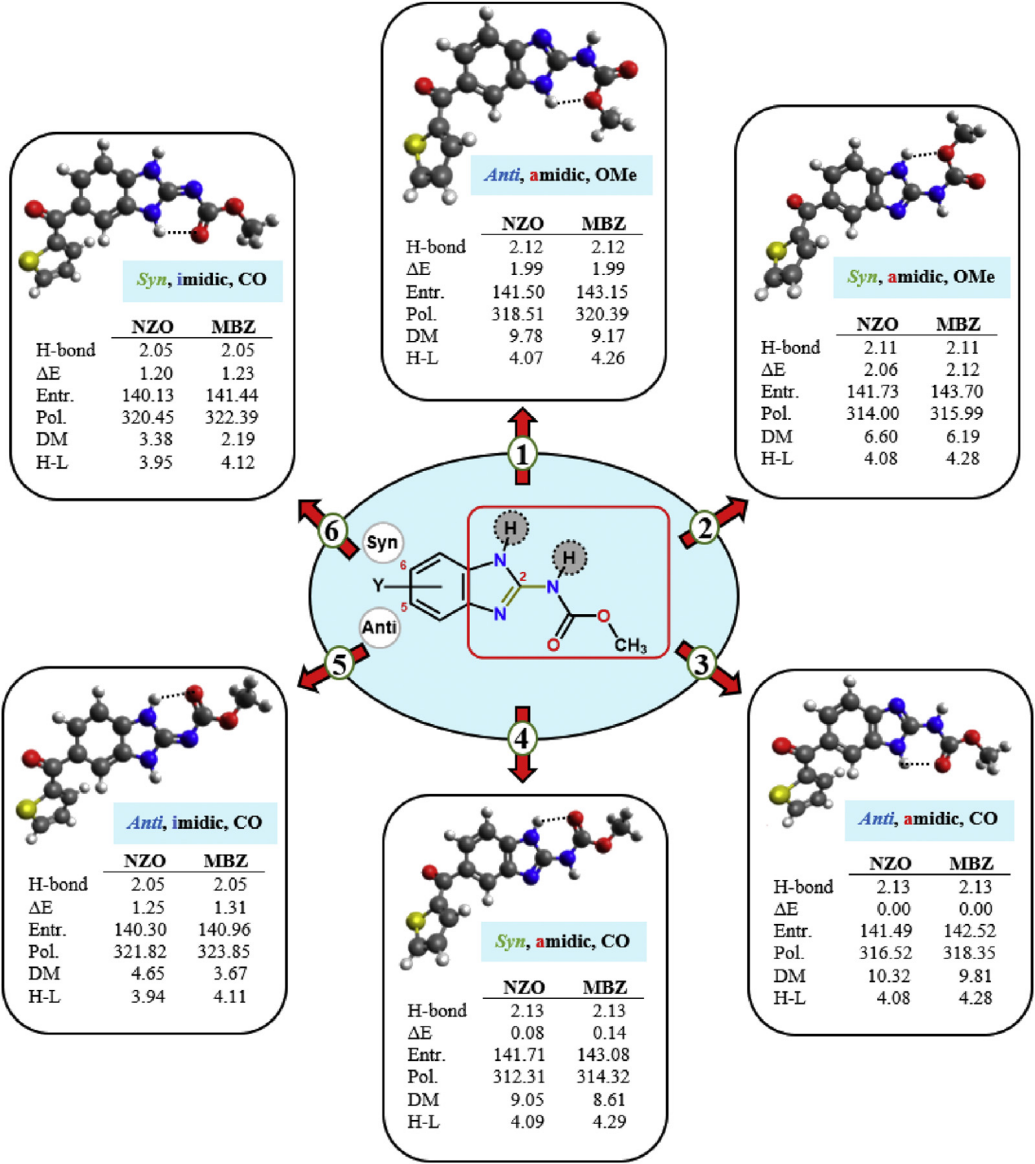
A representative structure of carbendazim-bearing drug where “Y” denotes to aliphatic or aromatic substitution at 5 and/or 6 position on benzimidazole moiety. Energy-optimized 3D structures of Nocodazole in water using B3LYP/6-311+G∗ model. Conformers are characterized based on 1) stereochemistry (*Anti* or *Syn*) between 5 (6)-substitution and the hydrogen of carbamate nitrogen (str.1–4) or oxygen of carbamate moiety (str. 5,6); 2) connection of benzimidazole nucleus with carbamate moiety through amidic or imidic bond; 3) intramolecular H-bond formation between hydrogen of benzimidazole nitrogen and oxygen of the methoxy group (OMe) or carbonyl group (CO). Dashed lines refer to H-bonding (in Å) between benzimidazolyl hydrogen and adjacent oxygen. NZO and MBZ denotes Nocodazole and Mebendazole respectively. ΔE (in kcal/mol) refers to change in energy relative to the most stable conformer (str. 3), where E is the electronic energy including zero-point energy correction. Entr. refers to entropy (in cal/mol-kelvin), Pol. Refers to polarizability (in a.u.), DM refers to dipole moment (in debye), and H-L refers to HOMO-LUMO energy gap (in eV).

The 2-carbamate substituent on the benzimidazole nucleus of Nocodazole was shown to adopt at least six stable configurations upon freezing the bond connecting the 5 (6)-substituent to the benzimidazole moiety during structural iterations, as can be seen in Figure 4. Four main features can be noted to distinguish the proposed 3D structures of Nocodazole: 1) whether the carbonyl oxygen is involved in formation of intramolecular hydrogen bonding with the benzimidazolyl hydrogen; 2) whether the carbamate group adopts a *syn* or *anti* configuration relative to substituent at 5 (6) position on benzimidazole ring; 3) whether the carbamate moiety is linked to the benzimidazole ring through amidic or imidic bond; and 4) whether the amide group atoms adopt a *cis* or *trans* orientation. The obtained preliminary data revealed existence of a small energy gap for interconversion of one conformer to another (<2.2 kcal/mol). Structure 3 was found the most stable conformer of Nocodazole as can be seen in Figure 5. While structure 2 was calculated as the least stable conformer, the energy difference between structures 1, 4, 5 and 6 and structure 3 were calculated at 1.99, 0.08, 1.25 and 1.20 kcal/mol respectively. In addition, the calculated structures showed distinct physicochemical patterns demonstrated through change in values of electronic energy, entropy, polarizability, and dipole moment. Values of some calculated parameters of each structure of Nocodazole (NZO) and Mebendazole (MBZ) are depicted in Figure 5.

### 3.2 Analysis of X-ray data giving more insights on conformational flexibility of Nocodazole and related derivatives

**Figure 6:**
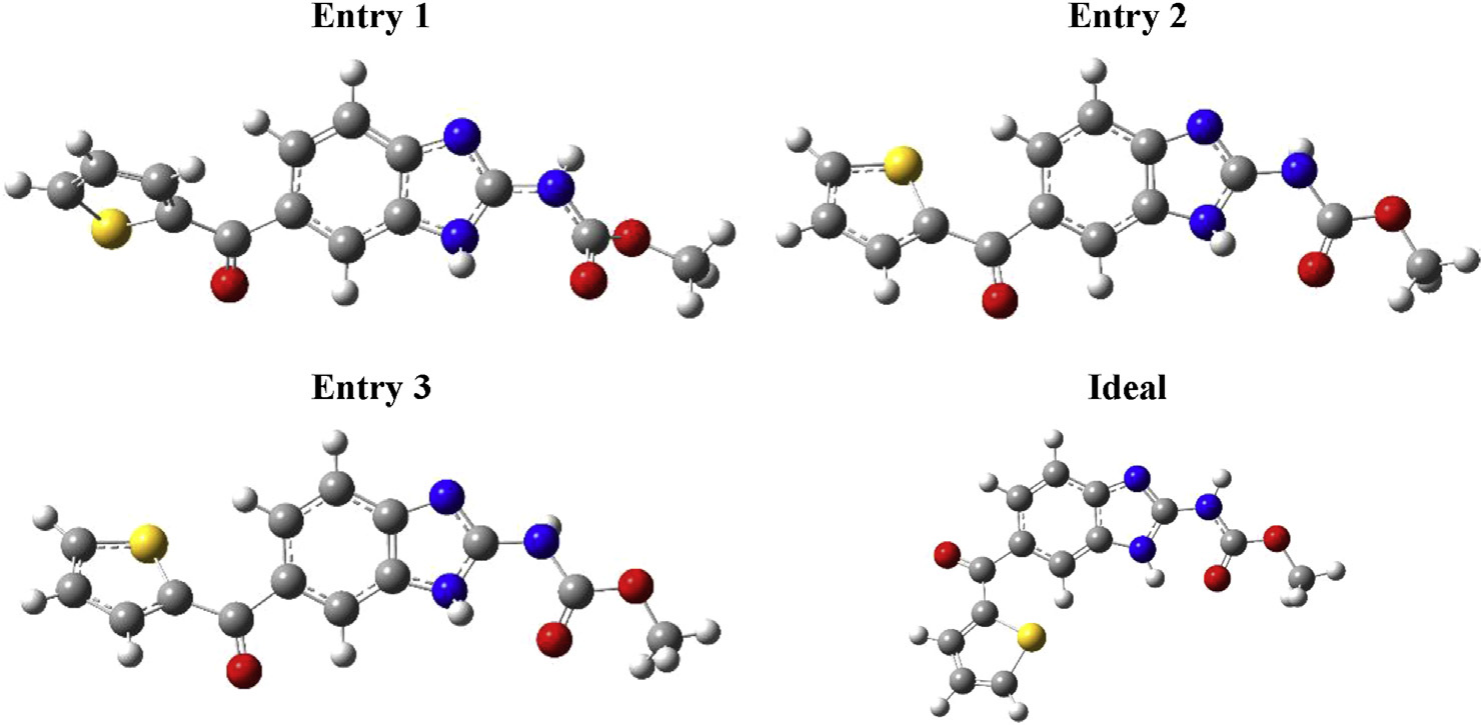
Shows the three entries of Nocodazole structure obtained from the Protein Data Bank (PDB) along with the ideal (calculated) structure.

The protein data bank (PDB) is a huge database comprising of the experimental X-ray data of some drug-protein complexes. PDB files contain values of the occupancy factor and temperature (Debye-Waller) factor which are descriptive parameters for examining the conformational flexibility of a drug and its binding site [36]. Occupancy factor can range from 0 to 1, where values closest to 1 indicate a precise positioning of atom in the crystal. While the temperature factor, also called B-factor, is used to quantify the uncertainty in atoms position in a crystal structure owing to static and/or dynamic disorders in crystal lattice. The static disorder is mainly due to different conformations of the drug in different unit cells of the protein, while the dynamic disorder originates from the atomic vibrations and translocations in the crystal [37, 38, 39]. By reviewing PDB database, we have found three entries (structures) for the deposition of Nocodazole with two different proteins as can be seen in Figure 6.

**Figure 7:**
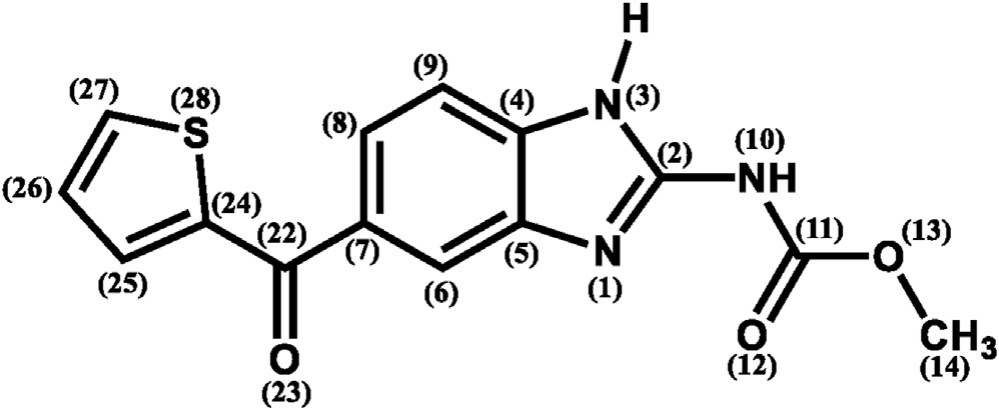
The 2D structure of Nocodazole displaying atoms number in consistency with data in Table 2.

**Figure 8:**
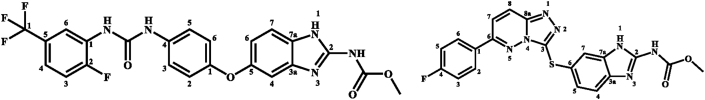
The 2D structure of methyl N-[5-[4-[[2-fluoro-5-(trifluoromethyl)phenyl]carbamoylamino] phenoxy]-1H-benzimidazol-2-yl]carbamate (PDB: GIG) (left panel) and methyl (6-{[6-(4-fluorophenyl)[1,2,4]triazolo [4,3-b]pyridazin-3-yl]sulfanyl}-1H-benzimidazol-2-yl)carbamate (PDB: 63B) (right panel). Atoms number are displayed in consistency with data in Table 3.

Although the X-ray crystal resolution for Nocodazole complexed with prostaglandin D (2) synthase (PDB: 3EE2) and the tubulin domain of T2R-TTL (PDB: 5CA1) was estimated at 1.91Å and 2.40Å respectively at temperature of 100K, it was noted that the Nocodazole molecule is more conformationally flexible within 3EE2 binding site than in 5CA1 pocket. That was evidenced since all non-hydrogen atoms show higher values of B-factor (>60 Å^2^) than with their counterparts in case of binding to 5CA1 (<50 Å^2^) as can be seen in Table 2. Interestingly, it was noticed that the sulphur atom has a high B-factor value for the three entries of Nocodazole at 63.70 Å^2^, 50.61 Å^2^, and 69.81 Å^2^ respectively. The uncertainty in allocating the position of sulphur atom in Nocodazole can be attributed to the formation of a hydrogen bond between sulphur atom and HO or HN or HS group on protein binding site of Nocodazole which can be essential for binding and exerting the biological activity. This trend was also noted with the carbonyl oxygen O (23) where B-factor values were obtained at 66.71 Å^2^, 40,66 Å^2^, and 50.29 Å^2^ respectively. In general, it was concluded that the carbonyl-sulphonyl substituent is more geometrically flexible than the carbendazimyl substituent. On the other hand, all Nocodazole atoms complexed with 3EE2 exhibited occupancy factor valued at 0.8 Å^2^, while having a value of 1 Å^2^ for Nocodazole bound with 5CA1. The values of occupancy factor of Nocodazole atoms demonstrates again the conformational flexibility of Nocodazole at 3EE2 binding site than in 5CA1 binding pocket.

**Table 2:** Temperature (B-factor) factor and occupancy factor values in (Å^2^) for non-hydrogen atoms of the three entries of Nocodazole (PDB: NZO) obtained from its X-ray cocrystal with prostaglandin D (2) synthase (PDB: 3EE2) and the tubulin domain of T2R-TTL (PDB: 5CA1). Atoms numbering can be viewed from Figure 7. Values in bold refer to thiophen sulphur and the adajcent carbonyl oxygen.

Entry-1 (3EE2)		Entry-2 (5CA1)		Entry-3 (5CA1)	
Atom	B-factor	Atom	B-factor	Atom	B-factor
O (12)	68.46	**S(28)**	**50.61**	**S(28)**	**69.81**
C (14)	68.42	**O(23)**	**40.66**	O (13)	54.51
O (13)	67.96	C (27)	35.50	**O(23)**	**50.29**
C (11)	67.90	C (24)	34.31	C (11)	45.73
**O(23)**	**66.71**	O (12)	33.11	N (3)	45.04
N (10)	66.08	C (22)	32.15	C (24)	42.80
N (1)	65.83	O (13)	32.09	C (27)	42.38
C (8)	65.81	C (7)	31.10	C (4)	42.23
C (9)	65.75	C (4)	31.06	O (12)	41.83
C (2)	65.70	C (26)	30.42	C (7)	41.46
C (7)	65.45	C (8)	30.09	N (10)	40.94
N (3)	65.41	C (9)	29.89	C (2)	40.40
C (27)	65.17	C (2)	29.20	C (22)	40.02
C (22)	65.10	N (1)	29.19	C (14)	39.18
C (4)	65.09	N (10)	28.12	C (26)	39.18
C (5)	64.80	C (14)	25.79	C (5)	39.08
C (6)	64.55	C (5)	25.34	C (8)	37.87
C (25)	64.02	C (11)	25.24	C (6)	35.32
**S(28)**	**63.70**	C (6)	25.00	N (1)	34.52
C (24)	63.67	N (3)	23.05	C (9)	33.01
C (26)	63.67	C (25)	14.24	C (25)	25.40
**Average**	65.68		30.29		41.95
**Occ. Factor**	0.8		1		1

X-ray data deposited in the protein data bank (PDB) for other derivatives of benzimidazole-2-carbamate, (PDB ID: GIG, 63B) -refer to Table 3 - showed that benzimidazole carbamates adopt configurations similar to that of Nocodazole structure 3 and 4 in Figures 4 and 5. Our theoretical computations are in excellent agreement with the reported X-ray data, since structures 3 and 4 are the two most stable (lowest energy) conformers of Nocodazole.

**Table 3:** Temperature (B-factor) and occupancy factor values in (Å^2^) for non-hydrogen atoms of methyl N-[5-[4-[[2-fluoro-5-(trifluoromethyl)phenyl]carbamoylamino]phenoxy]-1H-benzimidazol-2-yl]carbamate (PDB: GIG) (left panel) obtained from its X-ray cocrystal with VEGFR-2 (PDB: 2OH4) and methyl (6-{[6-(4-fluorophenyl)[1,2,4]triazolo [4,3-b]pyridazin-3-yl]sulfanyl}-1H-benzimidazol-2-yl)carbamate (PDB: 63B, 2 entries) (right panel) co-crystalized with Hepatocyte GFR (PDB: 5HNI). The 2D structures and atoms numbering can be viewed from Figure 8.

Methyl N-[5-[4-[[2-fluoro-5-(trifluoromethyl)phenyl] carbamoylamino] phenoxy]-1H-benzimidazol-2-yl] carbamate		Methyl (6-{[6-(4-fluorophenyl)[1,2,4] triazolo [4,3-b]pyridazin-3-yl]sulfanyl}-1H-benzimidazol-2-yl)carbamate	
Atom Number	B-factor	Atom Number	B-factor
C36	50.45	F	38.29
O35	46.86	F	37.08
F3	46.26	O2	36.90
F4	46.20	O1	36.83
F11	44.51	O1	36.41
F1	44.16	O2	36.07
C2	43.44	C17	35.62
O34	43.38	C17	35.19
C33	43.01	C20	34.98
C9	42.46	N4	34.80
C10	42.27	C14	34.78
C8	41.43	C14	34.75
C5	41.36	C15	34.58
N32	41.36	C2	34.18
C6	41.35	C19	34.13
C7	41.10	C19	33.95
N26	39.89	N3	33.94
N12	39.74	C10	33.84
C21	39.71	N1	33.60
C24	39.67	C20	33.59
C17	39.63	C11	33.40
C20	39.44	N2	33.36
C19	39.30	C7	33.22
C13	39.17	C1	33.16
C16	39.11	C15	33.13
C23	38.72	C3	33.11
O22	38.65	C4	33.08
C31	38.63	C6	32.89
C18	38.62	C10	32.88
C30	38.56	C7	31.76
N15	38.31	C11	31.73
C25	38.15	N4	31.47
C27	37.52	C4	30.88
N28	36.65	N3	30.76
O14	36.58	C2	30.68
C29	36.28	N7	30.51
		C1	30.36
		S1	30.26
		N1	30.21
		N2	30.06
		N7	29.69
		C6	29.60
		C3	29.41
		S1	28.48
		C5	27.38
		C8	26.47
		C5	26.31
		C18	25.76
		C8	24.61
		N5	24.42
		C9	24.41
		C12	24.24
		C18	24.07
		C9	23.78
		N6	23.70
		C16	23.66
		N5	23.54
		C12	23.01
		N6	22.99
		C13	22.94
		C13	22.89
		C16	22.54
**Average**	40.89		30.55
**Occ. Factor**	1		1

### 3.3 Simulated optical spectroscopy data

**Figure 9:**
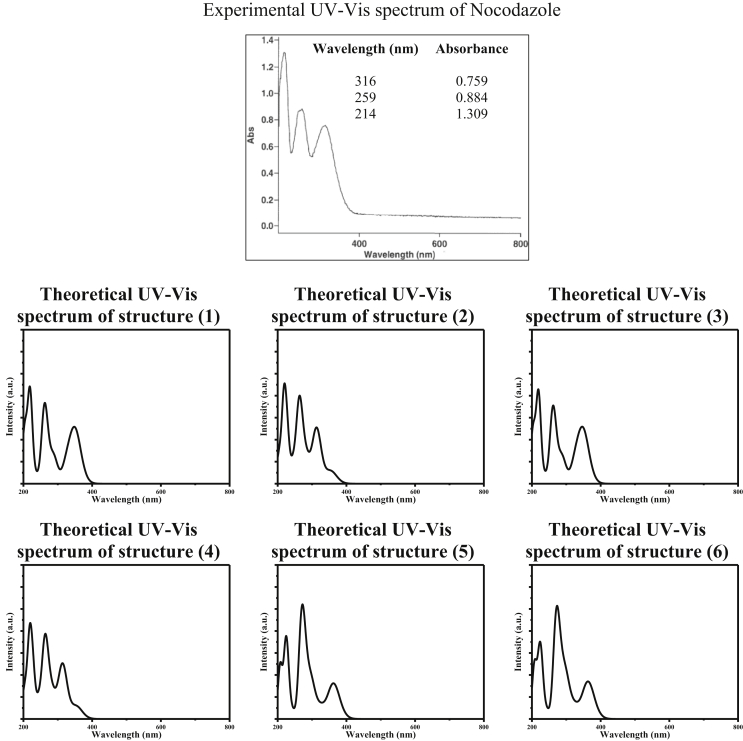
The experimental UV-Vis spectrum of Nocodazole in ethanol obtained from Cayman Chemical company. The inset table lists the values of maximum absorption peaks in nanometres along with the corresponding optical density (absorbance) in atomic units. Besides, the simulated UV-Vis spectra of 6 structures of Nocodazole in ethanol calculated at B3LYP/6-311+G∗.

By reviewing literature, we haven't found a reported UV-Vis spectrum nor electronic circular dichroism (ECD) spectrum for Nocodazole. Cayman Chemical company has provided us with the UV-Vis spectrum of the ethanolic solution of Nocodazole, refer to Figure 9. Nocodazole showed three prominent peaks at 316nm, 259nm, and 214nm with increasing optical density in order of decreasing wavelength of excitation light. The wavelength maxima obtained from the simulated UV-Vis spectra for the six studied structures are tabulated in Table 4. The simulated data revealed that structures (2) and (4) are in a good agreement with the experimental data which means structures (2) and (4) give -most probably- the most descriptive geometry for Nocodazole molecule. It is noteworthy mentioning that simulated spectra of six studied structures also showed the same trend where the optical density of absorption maximum peaks at shorter wavelengths are more enhanced than with absorption maxima beyond 300nm.

**Table 4:** The calculated excitation energy (absorption maximum) values of the studied 6 structures of Nocodazole in ethanol along with their corresponding oscillator strength values calculated at B3LYP/6-311+G∗.

Structure	(1)	(2)	(3)	(4)	(5)	(6)
Wavelength (nm)	328	313	328	314	300	301
Osc. Str.	0.1422	0.3351	0.1507	0.3330	0.1519	0.1828

Wavelength (nm)	261	263	261	262	270	270
Osc. Str.	0.4675	0.4651	0.4531	0.4465	0.5898	0.5324

Wavelength (nm)	219	219	219	219	221	221
Osc. Str.	0.2256	0.4108	0.2285	0.3881	0.1747	0.2151

**Figure 10:**
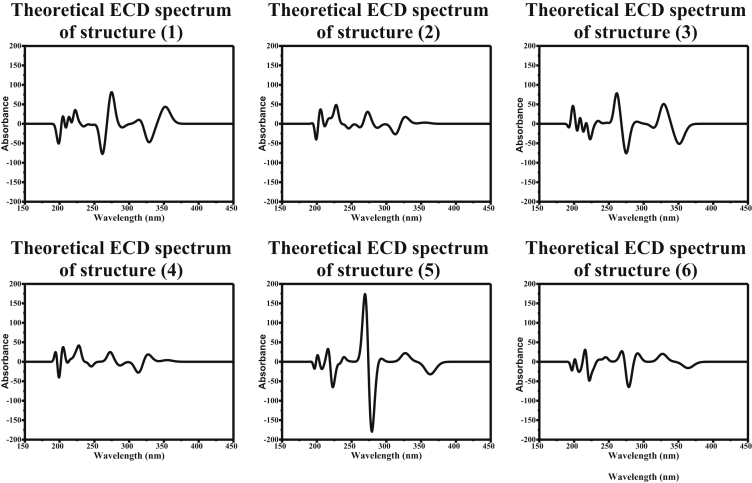
The calculated electronic circular dichroism (ECD) spectrum of the six studied structures of Nocodazole.

Electronic circular dichroism (ECD) measures the difference in absorbance of right- and left-circularly polarized light by a substance rather than the commonly used absorbance of isotropic light as in UV-Vis measurements [40]. We herein report the simulated ECD spectra of the six studied structures of Nocodazole as can be seen in Figure 10. It is obvious that each structure of Nocodazole exhibited a distinctive ECD spectrum since the change in Nocodazole conformation leaded to a change in chirality of the molecule. Interestingly, the ECD spectrum of structure (2) and (4) are nearly identical, even though their structure are completely different. This finding showcase the significance of combining the experimental data and theoretical calculation for structural characterization of molecules. By utilizing either of the two, researcher can end up with misinterpreted geometry of an active therapeutic molecule.

It is well known that the most energetically stable conformer of a drug is not necessary the most biologically potent form [41]. That is because not only the geometrical feature of a drug molecule is crucial for binding interaction with target protein, but also electronic configuration of a molecule plays an important role. The orbital charge density of the highest occupied molecular orbital (HOMO) and lowest unoccupied molecular orbital (LUMO) besides the two lower MO (HOMO-1 and HOMO-2) and the two higher MO (LUMO+1 and LUMO+2) of the studied six structures of Nocodazole are depicted in Tables 5 and 6. It was noted that the electron density are densely localized on thiophene moiety and to lesser extent on the benzimidazolyl core at LUMO, LUMO+1, and LUMO+2. While the electron density is significantly delocalized from the carbamate side chain of the molecule. In case of HOMO and HOMO-1, the electron density is mainly localized over benzimidazole core, however it is mainly localized over the thiophen group in case of HOMO-2. Since HOMO and LUMO are the main contributors to the binding interaction between a drug and target receptor, the obtained calculations postulate the occurrence of electron transfer from the benzimidazole core to the thiophen moiety upon binding. More experimental and theoretical studies should be conducted to confirm the charge transfer mechanism which is beyond the scope of current study.

**Table 5:** Charge density of the outermost molecular orbital of three studied structures of Nocodazole along with the map of electrostatic potential (MEP). The MO energies were calculated at B3LYP/6-311+G∗ level of theory.

	Structure (1)	Structure (2)	Structure (3)
LUMO+2			
LUMO+1			
LUMO			
HOMO			
HOMO-1			
HOMO-2			
MEP

**Table 6:** Charge density of the outermost molecular orbital of three studied structures of Nocodazole along with the map of electrostatic potential (MEP). The MO energies were calculated at B3LYP/6-311+G∗ level of theory.

	Structure (4)	Structure (5)	Structure (6)
LUMO+2			
LUMO+1			
LUMO			
HOMO			
HOMO-1			
HOMO-2			
MEP			

Molecular electrostatic potential (MEP) maps that quantify the electronic density distribution over the whole molecule are also depicted in Tables 5 and 6 (last row). The electron density changes from the more electronegative (red) to the more electropositive (blue). The MEP of the studied structures are quite different revealing that the change in drug conformation does result in a significant change in electronic configuration of the molecule. The electron density of MO and MEP of Nocodazole entries obtained from PDB are deposited in Table 7.

**Table 7:** Charge density of the outermost molecular orbital of three entries of Nocodazole structures obtained from PDB along with the map of electrostatic potential (MEP). The MO energies were calculated at B3LYP/6-311+G∗ level of theory.

	Entry-1	Entry-2	Entry-3
L+2			
L+1			
LUMO			
HOMO			
H-1			
H-2			
MEP			

Both of them -the structural and electronic configuration-are a crucial determinant of mechanism of binding interaction with target receptor, hence the biological activity that would be exerted by a drug. That is the foundation of drug repurposing strategies where the binding interactions of a drug which was abandoned with other protein receptors would be investigated. This is because the conformational flexibility of a drug molecule facilitates its binding interaction with multiple biological receptors.

Overall, the conducted studies emphasized that the use of a proper level of theory in performing theoretical calculations is crucial for obtaining an accurate prediction of structural geometry of a studied molecule. Moreover, theory can provide us with quantitative measurements of some of the physicochemical properties of a studied molecule that cannot be experimentally obtained such as bond length, bond angle, atomic charges, molecular orbital energy,….etc. Theory can also give information about binding affinity, binding interactions, binding energy,…etc between the drug and its target protein through molecular dynamic studies. Data obtained from theory gives predictive information about the structures, energies and charges of the free form of the drug (at atomic level) and of the drug-receptor complex (at molecular level), which is of great importance to understand pharmacodynamics of anticancer drugs and reasons behind development of cells resistance toward drugs which in turn help in development of more effective anticancer drugs less susceptible to development of drug resistance and/or repurposing drugs for use in oncology.

## 4 Conclusion

Carbendazim nucleus is an important conformationally flexible pharmacophore. Therefore various numbers of drug conformers can exist for a single drug molecule. By theoretically studying the spatial and electronic properties at the atomic and molecular levels, researchers can gain deep understanding of structural and electronic changes upon binding of a drug with target receptors. Which in turn will help in repurposing drugs and profiling drugs pharmacodynamics. By performing calculations on Nocodazole structures extracted from PDB, the structural and electronic features of different drug conformers were elucidated giving more insights for studying other carbendazim-bearing drugs.

## Declarations

### Author contribution statement

Muhammad Khattab: Conceived and designed the experiments; Performed the experiments; Analyzed and interpreted the data; Contributed reagents, materials, analysis tools or data; Wrote the paper.

### Funding statement

This research did not receive any specific grant from funding agencies in the public, commercial, or not-for-profit sectors.

### Competing interest statement

The authors declare no conflict of interest.

### Additional information

No additional information is available for this paper.
